# Best Prophylactic Strategy in Groups at Risk of Intraoperative Floppy Iris Syndrome Development: Comparison Between Atropine Instillation and Adrenaline Intracameral Injection

**DOI:** 10.2174/1874364101812010034

**Published:** 2018-03-30

**Authors:** Raffaele Nuzzi, Paolo Arnoffi, Federico Tridico

**Affiliations:** 1 Eye Clinic Section and Specialization School in Ophthalmology, Institute of Ophthalmology, Department of Surgical Sciences, University of Turin, Turin, Italy; 2 Opthalmology Division, S. Luigi Gonzaga University Hospital, Orbassano, Turin, Italy

**Keywords:** IFIS, Iris, Phacoemulsification, Tamsulosin, α1A antagonists, Mydriatic agents

## Abstract

**Background:**

Intraoperative Floppy Iris Syndrome (IFIS) is an important cause of surgical complications and iris defects in patients undergoing phacoemulsification that were treated with selective subtype α1A receptor antagonists for a long period of time. To date, no definitive preventive strategy has emerged, yet. The need of prophylaxis is dictated by the high prevalence of males affected by benign prostatic hyperplasia undergoing cataract surgery.

**Objective:**

To identify the best prophylactic strategy in groups at risk of IFIS development by comparing two mydriatic treatments in course of phacoemulsification surgery.

**Methods:**

81 eyes of 81 patients in treatment with Tamsulosin were enrolled in the study. 43 eyes were treated with atropine sulfate 1% while 38 eyes received an injection of mydriatic solution containing epinephrine in the anterior chamber. All phacoemulsifications were videotaped in order to assess the occurrence of IFIS and the severity of the syndrome.

**Results:**

The treatment group showed a statistically significant reduction (p = 0.0115) of floppy iris syndrome incidence, from 86.05% (37/43) of the atropine group to 60.53% (23/38). The analysis showed a reduction of IFIS mild form only, whereas the incidence of severe forms remained unchanged.

**Conclusions:**

We believe that IFIS may arise through two different mechanisms: pharmacological antagonism and anatomical modifications. Patients suffering from mild forms of the disease showed a statistically significant reduction of IFIS incidence after intraoperative prophylaxis due to epinephrine’s ability to displace Tamsulosin, resulting in the increase of iris tone when the disease is caused mainly by receptorial antagonism. On the contrary, prophylaxis does not deliver any valuable result in case of severe forms where the anatomical variations play a major role.

## 1 INTRODUCTION

The use of selective subtype α1A Receptor Antagonists (ARA α1A) (such as tamsulosin and silodosin) to treat Benign Prostatic Hyperplasia (BPH) has shown to reduce the hypotensive side effects of previous drugs (alfuzosin, doxazosin), increasing, however, the occurrence of ocular side effects [1].

Induced alterations become more evident during phacoemulsification procedures, leading to Intraoperative Floppy Iris Syndrome (IFIS). First described in 2005 by Chang and Campbell [2], IFIS is characterized by the presence of the classical triad consisting in fluctuation, miosis and progressive iris stroma prolapse through the surgical corneal tunnel, despite microincisions of 2.75, 2.2 or 1.8 mm. The clinical presentation may vary from mild to severe forms in which all three features occur [2].

The presence of IFIS often increases the risk of posterior capsule lens rupture with vitreous loss, lens nucleus displacement into the vitreous chamber, iris lacerations or atrophy and loss of ocular pigment, hyphema, and zonular disinsertion [2, 3].

The incidence of IFIS is about 0.5-2% in people who have never taken alphalitic drugs compared with 70% in those treated with alpha antagonists [4].

Extensive efforts have been made to identify the best preventive strategy [4]. To date, no definitive protocol (which has to be not only universally acknowledged but also standardized) has emerged, yet. The need of a preventive strategy is dictated by the high prevalence of males affected by benign prostatic hyperplasia undergoing cataract surgery. This problem is becoming more relevant also due to life expectancy elongation. Moreover, female subjects are not completely spared by this syndrome, since numerous other drugs including zuclopenthixol, risperidone, mianserin, chlorpromazine, quetiapine, labetalol and saw palmetto extract [5-7] were associated with IFIS, although less frequently. The main aim of this work is the comparison of the prophylactic efficacy of two mydriatic treatments, one that acts as a parasympatholytic (thus pupiloplegic) and the other based on the administration of an intracameral adrenergic agent.

## 2 MATERIAL AND METHODS

This study adheres to the principles of the Declaration of Helsinki and received the approval of the institutional ethics committee of the center where it was conducted.

Eighty-one eyes (from 81 male patients) under treatment with uninterrupted Tamsulosin (for at least 1 year) and affected by cataracts were enrolled in the study and enlisted for phacoemulsification surgery. Participants were subjected to preoperative ophthalmological evaluation including collection of personal data, ocular examination at the slit lamp, fundus examination after pharmacological mydriasis, acquisition of keratometric values with Javal ophthalmometry, acquisition of corneal topography data with Oculus Pentacam (with collection of central corneal thickness values, anterior chamber depth and iridocorneal angle width), intraocular pressure measurement with Goldman applanation tonometry, execution of ocular biometry with ultrasound and optical methods, manifest refraction measurement, uncorrected and best-corrected visual acuity examination.

43 patients were treated with treatment pattern A and 38 patients with the pattern B. Patients with pseudoexfoliation syndrome, miotic diabetic pupil, chronic use of miotic drugs, were excluded from the study as well as those with a history of α1 adrenergic receptor antagonist intake other than tamsulosin.

All patients received an ocular mydriatic insert (tropicamide/phenylephrine 0.28/5.4 mg) placed in the conjunctival sac 1 hour before surgery. In addition, Group A received atropine sulfate 1% instillation at 40 and 20 minutes before surgery, while Group B received an injection of a mydriatic solution in the Anterior Chamber (AC) at the beginning of surgery.

Group B’s solution, similar to the epi-shugarcaine formulated by Shugar [8], featured 2% lidocaine, adrenaline 1mg/ml without bisulfites and ophthalmic balanced salt solution (BSS Plus) at the following concentrations: epinephrine 1:3000 in a solution composed of lidocaine 2:5 and BSS Plus. Sulfite-free epinephrine was used to avoid the risk of endothelial damage as reported in Liou’s studies [9].

The BSS Plus was preferred to normal BSS because the pH of the solution, at the above-mentioned concentrations, did not meet the safety criteria set out by Gonnering (the mean pH out of 6 preparations was 6.05, related to regular BSS, and 7.04, if related to BSS plus) [10].

The solution was prepared in the operating room 10 minutes before use and the surgeon injected from 0.5 to 0.6 cc in front of the iris after performing the corneal incision. After 15-20 seconds an ophthalmic viscosurgical device (OVD) was injected (in our study 4% chondroitin sulfate, 3% hyaluronate) and surgery proceeded with regular procedure.

All 81 phacoemulsifications were performed by the same surgeon using the same technique and equipment (phacoemulsificator Bausch & Lomb STELLARIS), and videotaped to assess the occurrence of IFIS and the severity of the syndrome in accordance with Chang and Campbell criteria [11] referring as absent (stable normal iris), mild (noticeable iris billowing without significant miosis or iris prolapse) moderate (iris billowing accompanied by either iris prolapse or pupil diameter reduction ≥2 mm) or severe form (iris billowing accompanied by iris prolapse with pupil diameter reduction ≥2 mm). A blinded reviewer performed IFIS grading for each video.

Blood pressure and heart rate were measured five minutes before surgery and 5 minutes after the beginning of the procedure.

Six months after surgery Best Corrected Visual Acuity (BCVA) was measured and any adverse event recorded. Potential biases such as age, diabetes, glaucoma, high blood pressure, cataract entity, ocular axial length, iridocorneal angle, AC depth and iris color were checked.

In data analysis, the significance of differences between proportions was tested using the Chi square test or Fisher Exact Test if needed, while the significance of differences for other quantitative data was tested using Student’s t-test if the distribution was normal and Mann–Whitney–Wilcoxon test in case of non-parametric values.

## 3 RESULTS

No statistically significant differences in the distribution of variables (age, nuclear density, ocular axial length, iridocorneal angle, anterior chamber depth, diabetes, hypertension, macular degeneration, glaucoma, iris color) were observed between groups under study Tables **1** and **2**.

There was a total of 60 IFIS events (74%) in patients taking tamsulosin. Group B showed a statistically significant difference (p = 0.0115) of floppy iris syndrome incidence - 60.53% (23/38 patients) - when compared with Group A - 86.05% (37/43 patients). Table **3** compares treatment group with syndrome severity.

Systolic Blood Pressure (SBP) and Heart Rate (HR) showed no significant differences in the study groups indicating the systemic safety of intracameral epinephrine at doses 1:3000.

Postoperative complications occurred in 33% (12 out of 36 subjects) of severe IFIS cases (44% of overall participants), whereas none of the subjects who developed mild or moderate IFIS suffered from postoperative complications. The main observed complication was iris subatrophy due to iris trauma following prolapse and repositioning maneuvers (8 cases in group A and 3 in group B, p = 0.28), while one subject from group B suffered from postoperative iris defect (p = 0.44).

All patients achieved a BCVA of 20/25 minimum, regardless of the mydriatic treatment used and IFIS severity.

## 4 DISCUSSION

Phacoemulsification is one of the most performed surgical operations and the number of males with benign prostatic hyperplasia needing cataract surgery is increasing due to life expectancy elongation. Tamsulosin is the most used receptor antagonist due to its α1A receptor selectivity but it is also the most common cause of IFIS, hence the interest in developing preventive measures.

This study was designed to evaluate the efficacy of two prophylactic treatments for IFIS in people treated with tamsulosin, focusing on the application of a drug stimulating the activity of iris dilator muscle in comparison with a pharmacologic block of iris constrictor muscle. In our study, 1% atropine was used as the parasympatholytic agent while the mydriatic solution conceived for this study – in accordance to findings from the existing literature made by prominent supporters such as Schulze, Masket and Belani [12, 13] - has been adopted as the sympathomimetic agent.

The interesting finding of a statistically significant IFIS reduction (p = 0.0115), in Group B, especially for mild forms, when treated with intracameral epinephrine 1:3000 becomes even more remarkable in the light of another consideration. In fact, this assessment is very encouraging for the development of a relatively unexplored and novel prophylaxis regimen in association with “rational” alpha-antagonist drug discontinuation. Given recent autoptic and in vivo evidence of iris dilator muscle thickness reduction, associated with denervation and fiber vacuolization in patients taking Tamsulosin [14, 15], we believe that IFIS may arise through two different mechanisms. In the early stages of treatment with selective ARAα1A, the cause of floppy iris would be related to the pharmacological receptor antagonism, whereas, after continuous medication use, the anatomical modifications become the predominant mechanism leading to incomplete resolution after treatment interruptions.

The significant reduction of IFIS mild forms can be explained by the epinephrine’s ability to displace Tamsulosin, leading to an increase of the iris tone, whereas the inefficacy of the mydriatic solution under evaluation in case of severe stages would be attributable to anatomical variations. The direct adrenaline activity on alpha1 receptor has therefore, greater effects in comparison with the indirect action of atropine. Therefore, the lower efficacy of atropine observed in this study may be due to its inability to displace Tamsulosin from the α1A receptor, since its mechanism of action is based on the inhibition of iris constrictor muscle activity.

Limits of the study are related to the relatively small sample size and lack of long-term prospective data, such as reports on postoperative complications at one year, in order to evaluate whether IFIS remains primarily a problem for ophthalmologists, who must deal with it in course of phacoemulsification surgery, and investigate eventual repercussions on the quality of patients’ vision.

Further evaluations would be useful to comprehend the timing of ARAα1A intake that leads to iris irreversible alterations in order to establish both prophylactic measures of tamsulosin suspension and/or preoperative use of adrenergic agents, limiting techniques of mechanical dilation (such as iris hooks) when irreversible anatomical damage has occurred.

Patients should be addressed to ophthalmological evaluation before IPB treatment prescription, especially in patients complaining a decreased bilateral vision due to cataracts.

In the light of an interdisciplinary protocol, attention should be focused also to common alpha-1 antagonist drugs prescribed by cardiologists and psychiatrists, since antagonist activity on the α1 receptor may be the main cause of IFIS also in women.

We believe that more attention should be given to prevention, rather than prophylaxis, through the establishment of an interdisciplinary collaboration with urologists and general practitioners. The former would treat benign prostatic hypertrophy with non-selective ARA α1 drugs (when possible), since it is reported by numerous studies, such as the one by Chang and Campbell [11], to cause lesser IFIS occurrence. This would leave Tamsulosin to be prescribed after the conclusion of the ophthalmological surgical process (cataract extraction in both eyes) and general practitioners should act upon both urologists’ and ophthalmologists’ supervision.

## CONCLUSION

This study confirms the ocular and systemic safety of sulphite free epinephrine 1:3000 buffered in BSS Plus and how IFIS does not impact on visual results if handled by experienced surgeons (since all patients achieved a BCVA minimum of 20/25). The mydriatic intracameral solution containing epinephrine was more effective than atropine in preventing IFIS in its mild form, but not in severe forms in which anatomical changes have occurred. According to data obtained, the use of intraoperative adrenergic agents would be ineffective in IFIS progression “containment” when anatomical alterations are suspected.

Given these considerations, the widespread opinion on the futility of ARAα1A withdrawal may be reconsidered, leading to a rational interruption in a subset of patients.

## Figures and Tables

**Table 1:** Quantitative participants’ data.

**Variable**	**Mean ± SD**		**p-value (95% CI)**
	**A** Group	**B** Group	
**Age (y)**	74,8 ± 6,7	73,4 ± 6	0.328 (-1.43 – 4.23)
**Nuclear density^1^**	13 ± 3	11,8 ± 2	0.040 (0.06 – 2.34)
**Ocular Axial lenght^2^ (mm)**	22,9^(4)^ ± 0,5	22,9^(5)^ ± 0,6	1.000 (-0.24 – 0.24)
**Iridocorneal angle^3^ (°)**	37 ± 10	31,3 ± 7,6	0.005 (1.73 – 9.67)
**A.C^6^. depth^2^ (mm)**	2,8 ± 0,6	2,6 ± 0,3	0.067 (-0.01 – 0.41)

**^1^ measured with “Pentacam Nucleus Staging” (Oculus Petacam).** **^2^ measured with Optical biometry (ZEISS IOL MASTER)** **^3^ measured with Pentacam HR -Oculus-.** **^4^ three patients data lost** **^5^ four patients data lost** **^6^ A.C. anterior chamber**

**Table 2:** Qualitative participants’ data.

**Variable**
	**A** Group n/total (%)	**B** Group n/total (%)	**p-value**
**Diabetes**	13/43 (30.23%)	11/38 (28.95%)	1.000
**Hypertension**	35/43 (81.39%)	28/38 (73.68%)	0.435
**Maculopathy**	9/43 (20.93%)	9/38 (23.68%)	0.795
**Glaucoma**	2/43 (4.65%)	1/38 (2.63%)	1.000
**Iris Color** **light** **dark**	11/43 (25.58%) 32/43 (74.41%)	9/38 (23.68%) 29/38 (76.32%)	1.000 1.000
**Lenght of tamsulosin intake at recruitment time**			
**1-3 years**	15/43 (34.88%)	15/38 (39.47%)	0.818
**3-5 years**	6/43 (13.95%)	4/38 (10.53%)	0.743
**>5 years**	22/43 (51.16%)	18/38 (47.37%)	0.825

**Table 3:** IFIS severity related to prophylactic treatment.

**IFIS**	**A Group** **n/N (%)**	**B Group** **n/N (%)**	**p-value**
**None**	6/43 (13.95%)	15/38 (39.47%)	0.0115
**Mild**	15/43 (34.8%)	6/38 (15.79%)	0.0748
**Moderate**	2/43 (4.65%)	1/38 (2.63%)	1.0000
**Severe**	20/43 (46.51%)	16/38 (42.11%)	0.8232
**Overall IFIS cases**	37/43 (86.05%)	23/38 (60.53%)	0.0115

## ABBREVIATION LIST

ARA α1A: = α1A Receptor Antagonists
BPH: = Benign Prostatic Hyperplasia
IFIS: = Intraoperative Floppy Iris Syndrome
AC: = Anterior Chamber
BSS: = Balanced Salt Solution
OVD: = Ophthalmic Viscosurgical Device
BCVA: = Best-Corrected Visual Acuity
SBP: = Systolic Blood Pressure
HR: = Heart Rate
